# Prevalence of Chest Pain, Depression, Somatization, Anxiety, Global Distress, and Substance Use among Cardiac and Pulmonary Rehabilitation Patients

**DOI:** 10.1155/2012/138680

**Published:** 2012-11-01

**Authors:** Eva R. Serber, Shenelle A. Edwards-Hampton, Brooke Yeager, Mark Clair, Marian Taylor, Sarah K. Galloway, Wendy E. Balliet, Alok Madan, Jeffrey J. Borckardt

**Affiliations:** ^1^Division of Bio-Behavioral Medicine, Department of Psychiatry and Behavioral Sciences, Medical University of South Carolina, Charleston, SC 29425, USA; ^2^Cardiopulmonary Rehabilitation, Medical University of South Carolina, Charleston, SC 29425, USA; ^3^Division of Cardiology, Department of Medicine, Medical University of South Carolina, Charleston, SC 29425, USA

## Abstract

Psychosocial factors of cardiovascular disease receive a preponderance of attention. Little attention is paid to psychosocial factors of pulmonary disease. This paper sought to describe psychosocial characteristics and to identify differences between cardiac and pulmonary patients entering a phase II rehabilitation program. Parametric and nonparametric analyses were conducted to examine scores on the Brief Symptom Inventory-18 (BSI-18) and the CAGE-D, administered at entry as standard clinical care. Participants were 163 cardiac and 63 pulmonary patients. Scores on the BSI-18 “chest pain” item indicated that more cardiac patients report chest pain than pulmonary patients. Among all subjects, chest pain ratings were positively related to anxiety, depression, and global distress. There were equivocal proportions of anxiety and somatization in patient groups. Pulmonary patients were more likely to endorse clinically significant levels of depression and global psychological distress than cardiac patients. Cardiac patients were significantly more likely to screen positively on the CAGE-D than pulmonary patients. Findings show a relationship between symptoms of chest pain and psychological distress. Despite equivalent proportions of anxiety and somatization between groups, a greater proportion of pulmonary patients reported symptoms of depression and global psychological distress, while more cardiac patients reported chest pain. Further research is needed to examine this paradigm.

## 1. Introduction

Cardiovascular disease (CVD) continues to be the most frequent cause of death worldwide, and close behind, chronic obstructive pulmonary disease (COPD) is estimated as the future third leading cause of death worldwide by 2030 [1]. A great deal of research exists related to behavioral and psychosocial variables in the pathogenesis and expression of CVD, as well as precipitating cardiac events [2, 3]; however, little information is available related to these variables in pulmonary disease.

Cardiac rehabilitation is a well-known, comprehensive, secondary prevention program that has been proven to reduce morbidity and mortality and improve quality of life in patients with CVD [4–7]. Studies have also shown an even greater reduction in mortality in patients with high psychosocial stress or depression who have improved their physical fitness and/or completed a cardiac rehabilitation program, while also reducing psychosocial stress and depression prevalence [6, 8]. Participation in cardiac rehabilitation consistently yields improved lipid profiles, exercise capacity, physical fitness, health behaviors, and psychological outcomes in both younger and older cardiac patients [5, 6, 9–12].

The multidisciplinary secondary prevention program of cardiac rehabilitation, a model of integrative care, is applied to other populations, including pulmonary patients, demonstrating to be an equally effective treatment. Cardiac and pulmonary diseases have several similar physical complaints (i.e., dyspnea, exercise intolerance, fluid retention, chest tightness, and heart palpitations), as well as psychosocial variables (i.e., depression, anxiety, substance use, somatization, social support, dietary choices, and level of patient education) that play a significant role in the etiology and course of both disease processes. However, pulmonary rehabilitation appears to be even more underutilized than cardiac rehabilitation [13, 14]. The research is also significantly less extensive, but demonstrates that pulmonary rehabilitation provides multiple benefits such as improving quality of life and exercise capacity and reducing dyspnea and symptoms of depression and anxiety [15–17].

Pulmonary patients who are referred to rehabilitative treatment are often provided with similar treatment and are often housed within or alongside a cardiac rehabilitation program. To our knowledge, there is no research that has explored similarities and differences of psychosocial variables in cardiac compared to pulmonary rehabilitation patients. Increased understanding of the different psychosocial strengths and challenges that cardiac and pulmonary patients face holds promise for identifying the unique needs of these patient populations and for the development of individualized treatment plans.

The current study sought to examine the psychosocial and behavioral characteristics in patients participating in cardiac and pulmonary rehabilitation programs. Given the similarities between cardiac and pulmonary patients, it was hypothesized that there would be no significant difference between self-reported symptoms of depression, anxiety, somatization, global distress, or substance abuse among cardiac and pulmonary patients.

## 2. Methods

In the present study, participants were patients initiating cardiac rehabilitation or pulmonary rehabilitation at a large academic medical center. As part of standard clinical care, patients were given various questionnaires regarding medical, psychosocial, and health behavior history. Patients complete their intake forms in a private waiting area while waiting for their initial consultation with a rehabilitation specialist. All patients used a web-based computer psychosocial screening system to complete the measures on-site at the facility. Rehabilitation staff was available to help patients log-in to the system and to assist with completion of the online questionnaires if necessary.

In this paper, we examined data obtained from the Brief Symptom Inventory-18 and the CAGE-D. IRB approval was obtained in order to report the data in aggregate for the purpose of this study.

### 2.1. The Brief Symptom Inventory-18 (BSI-18) [18]

The BSI-18 includes 18 symptoms; participants rate how much their level of distress over the past seven days using a five-point Likert scale (0 “not at all” to 4 “extremely”). It includes three subscales (depression, anxiety, somatic; range 0–24) and a total score (range 0–72). Item number 4 (chest pain) loads on the somatic subscale. Higher scores indicate more distress. This measure has been validated with various community and medical samples [18].

### 2.2. CAGE Questionnaire [6]

CAGE is a mnemonic for assessing: cutting down, annoyance by criticism, guilty feeling, and eye-openers. The CAGE questionnaire was used to screen for substance use (CAGE-D) [6].

### 2.3. Planned Analyses

Descriptive statistics were used to describe the sociodemographic and psychosocial characteristics of the sample. Independent sample *t* tests or Pearson *r* chi-squared (*χ* ^2^) tests were conducted, depending on continuous or categorical variables, to examine whether there were differences between cardiac and pulmonary patients. For all analyses, significance was set at *α* = .05.

## 3. Results

Completing the two screening questionnaires (BSI-18 and CAGE-D) were a total of 226 patients. There were 163 cardiac (*M* age = 61 ± 11, 62% male and married) and 63 pulmonary (*M* age = 67 ± 12, 59% male and 67% married) patients. There were significant differences between cardiac and pulmonary patients on sociodemographic variables. Cardiac patients were more likely to be male compared to pulmonary patients (61.8% versus 41.2%, *P* = .04), whereas pulmonary patients were more likely to be older than cardiac patients (*M* age = 61 ± 11 versus *M* age = 67 ± 12, *P* < .001).

Examination of the endorsement of the “chest pain” item (Item number 4) on the BSI-18, demonstrated that 22.2% of cardiac patients reported moderate to severe pain compared to 14.6% of pulmonary patients. However, there was no significant difference in mean chest pain ratings between groups (*t*(224) = 1.57, *P* = .12). Among all subjects, chest pain intensity ratings were positively related to anxiety (*r*(226) = .29, *P* < .001), depression (*r*(226) = .21, *P* = .002), and global distress (*r*(226) = .44, *P* < .0001). Correlation was expected and does not exceed the collinearity cutoff of *r* = .70 [19].

No difference was found in the incidence of somatization between cardiac (19.7%) and pulmonary (21.1%) rehabilitation patients (*χ* ^2^ *P* = .08), nor in the incidence of clinically significant anxiety (6.4% for cardiac patients; 8.9% for pulmonary patients; *χ* ^2^ *P* = .61). However, pulmonary rehabilitation patients were significantly more likely to exceed the cut-off for clinically significant depression (15.6%) than cardiac patients (7.7%; *χ* ^2^ = 4.46, *P* = .03), and for global psychological distress (17.8% for pulmonary patients; 9.4% for cardiac patients; *χ* ^2^ = 4.41, *P* = .03). However, cardiac patients were significantly more likely to screen positively on the CAGE-D (13.8%) than pulmonary patients (5.4%; *χ* ^2^ = 4.62, *P* = .02) for substance abuse.

## 4. Discussion

In the current sample of cardiac and pulmonary patients initiating a phase II rehabilitation program, symptoms of chest pain were commonly reported. Ratings of pain as moderate to severe were more commonly endorsed by cardiac patients compared to pulmonary patients. There were not significant differences between cardiac and pulmonary patients on incidence of somatization or anxiety. This is notable in that chest pain is a commonly reported somatization and anxiety symptom. Also seen in these cardiac patients at entry into rehabilitation program, was a higher probability of substance abuse, as measured by the CAGE-D questionnaire compared to pulmonary patients. Pulmonary patients, however, were more likely to have clinically significant depression and global psychological distress than were cardiac patients.

Both patient populations reported similar amounts of physical complaints (somatization). Interestingly, many of the medical symptoms associated with chest pain in cardiac and pulmonary patients (e.g., shortness of breath, fatigue) are physiological symptoms that overlap with anxiety and depression. Given that both patient groups in this study reported similar levels of somatization, higher reports of physical pain by cardiac patients compared to pulmonary patients may reflect differences in how psychological distress manifest within these different patient samples. Increased report of chest pain in cardiac patients with anxiety is commonly seen as patients who become hypervigilant to their somatic symptoms, particularly those that may be construed as cardiac in origin [20, 21]. Current findings may also suggest that these cardiac patients are more likely to misuse substances as a means of coping with and reducing distressing physiological responses, such as cardiac and noncardiac chest pain.

Compared to the cardiac patients, these pulmonary patients, on the other hand, indicate that the distress symptoms they are experiencing are less related to physiological pain compared to cardiac patients. Given the higher probability of pulmonary patients endorsing depression and global psychological distress, it is critical to identify the specific psychosocial and somatic symptoms pulmonary patients express when experiencing psychological difficulties. Particularly highlighted in the current sample, patients' report of distress symptomatology is missed, under-recognized and -appreciated; and therefore, not treated adequately.

## 5. Limitations

The findings need to take into account several limitations, including the minimal number of data variables available for examination, and the cross-sectional, observational nature of the study, as the data was obtained as part of clinical care and not a formal research study. It would be advantageous for a future study to examine the comprehensive intake and exit data, as well as change in outcomes over time. The current sample was relatively homogenous, characterized largely by Caucasian, middle-aged and married patients. Accordingly, generalizability of findings may be somewhat limited.

## 6. Conclusion

Findings from the current study offer valuable insight into ways in which rehabilitative treatment can be tailored to meet the unique needs of cardiac and pulmonary patients. While more is known about cardiac patients presenting to phase II rehabilitation programs, a considerable proportion of both cardiac and pulmonary patients present with an array of psychosocial, health behavior, and physical concerns such as depression, anxiety, pain, and substance use and misuse [22–24]. Differences between patient groups have both assessment and treatment implications.

## References

[1] Fabbri LM, Luppi F, Beghé B, Rabe KF (2008). Complex chronic comorbidities of COPD. *European Respiratory Journal*.

[2] Rozanski A, Blumenthal JA, Davidson KW, Saab PG, Kubzansky L (2005). The epidemiology, pathophysiology, and management of psychosocial risk factors in cardiac practice: the emerging field of behavioral cardiology. *Journal of the American College of Cardiology*.

[3] Dimsdale JE (2008). Psychological stress and cardiovascular disease. *Journal of the American College of Cardiology*.

[4] O'Connor GT, Buring JE, Yusuf S (1989). An overview of randomized trials of rehabilitation with exercise after myocardial infarction. *Circulation*.

[5] Taylor RS, Brown A, Ebrahim S (2004). Exercise-based rehabilitation for patients with coronary heart disease: systematic review and meta-analysis of randomized controlled trials. *American Journal of Medicine*.

[6] Lavie CJ, Thomas RJ, Squires RW, Allison TG, Milani RV (2009). Exercise training and cardiac rehabilitation in primary and secondary prevention of coronary heart disease. *Mayo Clinic Proceedings*.

[7] Hammill BG, Curtis LH, Schulman KA, Whellan DJ (2010). Relationship between cardiac rehabilitation and long-term risks of death and myocardial infarction among elderly medicare beneficiaries. *Circulation*.

[8] Milani RV, Lavie CJ (2007). Impact of cardiac rehabilitation on depression and its associated mortality. *American Journal of Medicine*.

[9] Benzer W, Platter M, Oldridge NB (2007). Short-term patient-reported outcomes after different exercise-based cardiac rehabilitation programmes. *European Journal of Cardiovascular Prevention and Rehabilitation*.

[10] Lavie CJ, Milani RV (2011). Cardiac rehabilitation and exercise training in secondary coronary heart disease prevention. *Progress in Cardiovascular Diseases*.

[11] Lavie CJ, Milani RV, O’Keefe JH, Lavie TJ (2011). Impact of exercise training on psychological risk factors. *Progress in Cardiovascular Diseases*.

[12] Lavie CJ, Milani RV (2006). Adverse psychological and coronary risk profiles in young patients with coronary artery disease and benefits of formal cardiac rehabilitation. *Archives of Internal Medicine*.

[13] Cafarella PA, Effing TW, Usmani ZA, Frith PA (2012). Treatments for anxiety and depression in patients with chronic obstructive pulmonary disease: a literature review. *Respirology*.

[14] Gelberg J, McIvor RA (2010). Overcoming gaps in the management of chronic obstructive pulmonary disease in older patients: new insights. *Drugs and Aging*.

[15] Coventry PA (2009). Does pulmonary rehabilitation reduce anxiety and depression in chronic obstructive pulmonary disease?. *Current Opinion in Pulmonary Medicine*.

[16] Coventry PA, Hind D (2007). Comprehensive pulmonary rehabilitation for anxiety and depression in adults with chronic obstructive pulmonary disease: systematic review and meta-analysis. *Journal of Psychosomatic Research*.

[17] Troosters T, Casaburi R, Gosselink R, Decramer M (2005). Pulmonary rehabilitation in chronic obstructive pulmonary disease. *American Journal of Respiratory and Critical Care Medicine*.

[18] Derogatis L (2000). *Brief Symptom Inventory (BSI) 18: Administration, Scoring, and Procedures Manual*.

[19] Kleinbaum DG, Kupper LL, Muller KE, Nizam A (1998). *Applied Regression Analysis and other Multivariable Methods*.

[20] White KS, Craft JM, Gervino EV (2010). Anxiety and hypervigilance to cardiopulmonary sensations in non-cardiac chest pain patients with and without psychiatric disorders. *Behaviour Research and Therapy*.

[21] Zvolensky MJ, Eifert GH, Feldner MT, Leen-Feldner E (2003). Heart-focused anxiety and chest pain in postangiography medical patients. *Journal of Behavioral Medicine*.

[22] Serber ER, Todaro JF, Tilkemeier PL, Niaura R (2009). Prevalence and characteristics of multiple psychiatric disorders in cardiac rehabilitation patients. *Journal of Cardiopulmonary Rehabilitation and Prevention*.

[23] de Voogd JN, Sanderman R, Postemaa K, van Sonderen E, Wempe JB (2011). Relationship between anxiety and dyspnea on exertion in patients with chronic obstructive pulmonary disease. *Anxiety, Stress and Coping*.

[24] Moullec G, Laurin C, Lavoie KL, Ninot G (2011). Effects of pulmonary rehabilitation on quality of life in chronic obstructive pulmonary disease patients. *Current Opinion in Pulmonary Medicine*.

